# Disease burden of stroke in rural South Africa: an estimate of incidence, mortality and disability adjusted life years

**DOI:** 10.1186/s12883-015-0311-7

**Published:** 2015-04-12

**Authors:** Mandy Maredza, Melanie Y Bertram, Stephen M Tollman

**Affiliations:** MRC/Wits Rural Public Health and Health Transitions Research Unit (Agincourt), School of Public Health, Faculty of Health Sciences, University of the Witwatersrand, Education Campus, St Andrews Road, Parktown, Johannesburg South Africa; World Health Organization, Geneva, Switzerland; Centre for Global Health Research, Umeå University, Umeå, Sweden; INDEPTH Network, Accra, Ghana

**Keywords:** Stroke, DALY, Incidence, Rural, South Africa, Agincourt health and socio-demographic surveillance site

## Abstract

**Background:**

In the context of an epidemiologic transition in South Africa, in which cardiovascular disease is increasing, little is known about the stroke burden, particularly morbidity in rural populations. Risk factors for stroke are high, with hypertension prevalence of more than 50%. Accurate, up-to-date information on disease burden is essential in planning health services for stroke management. This study estimates the burden of stroke in rural South Africa using the epidemiological parameters of incidence, mortality and disability adjusted life year (DALY) metric, a time-based measure that incorporates both mortality and morbidity.

**Methods:**

Data from the Agincourt health and socio-demographic surveillance system was utilised to calculate stroke mortality for the period 2007–2011. Dismod, an incidence-prevalence-mortality model, was used to estimate incidence and duration of disability in Agincourt sub-district and ‘mostly rural’ municipalities of South Africa. Using these values, burden of disease in years of life lost (YLL), years lived with disability (YLD) and DALYs was calculated for Agincourt sub-district.

**Results:**

Over 5 years, there were an estimated 842 incident cases of stroke in Agincourt sub-district, a crude stroke incidence rate of 244 per 100,000 person years. We estimate that 1,070 DALYs are lost due to stroke yearly. Of this, YLDs contributed 8.7% (3.5 – 10.5%) in sensitivity analysis). Crude stroke mortality was 114 per 100,000 person-years in 2007–11 in Agincourt sub-district. Burden of stroke in entire rural South Africa, a population of some 13,000,000 people, was high, with an estimated 33, 500 strokes occurring in 2011.

**Conclusions:**

This study provides the first estimates of stroke burden in terms of incidence, and disability in rural South Africa. High YLL and DALYs lost amongst the rural populations demand urgent measures for preventing and mitigating impacts of stroke. Longitudinal surveillance sites provide a platform through which a changing stroke burden can be monitored in rural South Africa.

**Electronic supplementary material:**

The online version of this article (doi:10.1186/s12883-015-0311-7) contains supplementary material, which is available to authorized users.

## Background

Stroke is the second-leading cause of death worldwide after ischaemic heart disease [1]. In 2010, stroke was responsible for 5.3 million deaths or 1 in 10 deaths worldwide. The absolute number of people affected by stroke has been increasing yearly since 1990, along with the numbers of disabled stroke survivors and deaths related to stroke [2]. It is estimated that, if current trends continue, by 2030 there will be 20 million annual stroke deaths and 70 million stroke survivors worldwide.

More than 80% of stroke burden occurs in low and middle-income countries (LMICs), yet reliable data on stroke epidemiology, particularly incidence and morbidity is scarce in these settings [3]. The paucity of data is even more pronounced in rural parts of Africa. This is a critical gap for a variety of reasons. Epidemiological data is required to better describe trends, and to develop appropriate cost-effective, prevention and treatment strategies. In the absence of up-to-date estimates on epidemiological burden of stroke, the economic impacts due to stroke are likely underestimated. Global estimates show that 3% of total health care system resources are devoted to stroke [4]. However, much of the data is derived from developed countries.

In South Africa (SA), stroke is responsible for some 25,000 deaths annually and 95,000 years lived with disability [5] yet a few published studies report on the epidemiology of stroke in rural parts of the country [6-8]. Whilst out-of-date, the evidence from these studies indicates that as far back as the 1990s, stroke was an important cause of death in rural South Africa. Analysis based on data for the years 1992–1995 indicated that stroke was responsible for 6% of total deaths in the Agincourt sub-district, rural North-Eastern South Africa [8]. A 2001 study, based on the same population, found a crude stroke prevalence of 243 per 100,000 [6], which is twice as high as rates reported for a rural Tanzanian district [9]. Recent evidence from an incidence-based study points to a high incidence of key risk factors for stroke in rural North-West Province. This study showed that a quarter (n = 84) of adults aged ≤35 years whose blood pressure was optimal at baseline in 2005, developed hypertension at 5 years of follow-up [10]. Similarly, consumption of sugar-sweetened beverages, doubled during the same period. Given the increase in risk factors and the implications for stroke, it is critical to understand the current burden of stroke in rural South Africa.

Empirical observation is the gold standard for obtaining epidemiological information. However, much of the available data in South Africa is on mortality with incidence and morbidity data almost lacking. Incidence-Prevalence-Mortality (IPM) models such as Dismod II can assist to fill this information gap by exploiting the causal structure of disease processes: incidence has to precede prevalence, and cause-specific mortality follows being diseased [11]. With at least 3 available input parameters such as case-fatality, remission, and prevalence the model can back-calculate incidence. In addition, these models allow one to check for the internal consistency of observations. A validation study of Dismod showed that provided past trends in disease epidemiology did not differ significantly from future trends, the model calculates correct results [11]. The Dismod models have been used in the global and national burden of disease studies to circumvent the challenge of incomplete data [12,13].

The primary aim of this paper is to contribute towards the practical understanding of stroke burden in rural South Africa. The analysis utilises population-based data from the Agincourt sub-district population of some 70,000 people, for the period 2007–2011, coupled with some modeling techniques [14]. Stroke incidence is assessed using Dismod II software, and total burden captured using the DALY metric. The DALY is a summary measure of population health that captures, into a single measure, both the mortality and severity of the morbidity associated with a disease. The DALY metric has been implemented in several studies including the Global Burden of Disease (GBD) studies and subsequent WHO updates [12,13,15] and the South African National Burden of Disease (SA NBD) Study of 2000 [16].To the authors’ knowledge, there are no published studies to-date that have examined the epidemiology of stroke in rural South Africa using the most recent available data on morbidity and mortality. Information generated from this research is intended to guide the planning of health services for stroke prevention and management.

## Methods

### Definition of a rural area

The term ‘rural’ suggests many contrasting images to people, such as agricultural landscapes, isolation, small towns, and low-population density [17]. We use the classification by Palmer Development Group (PDG), a public sector consulting firm which classifies rural areas as either ‘small towns’ or ‘mostly rural’ municipalities (Additional file 1: Table S2 and Table S3) [18]. The Agincourt sub-district falls under Bushbuckridge municipality ‘mostly rural’; in this study, ‘rural’ refers explicitly to such municipalities.

### Setting and population

**Figure 1:**
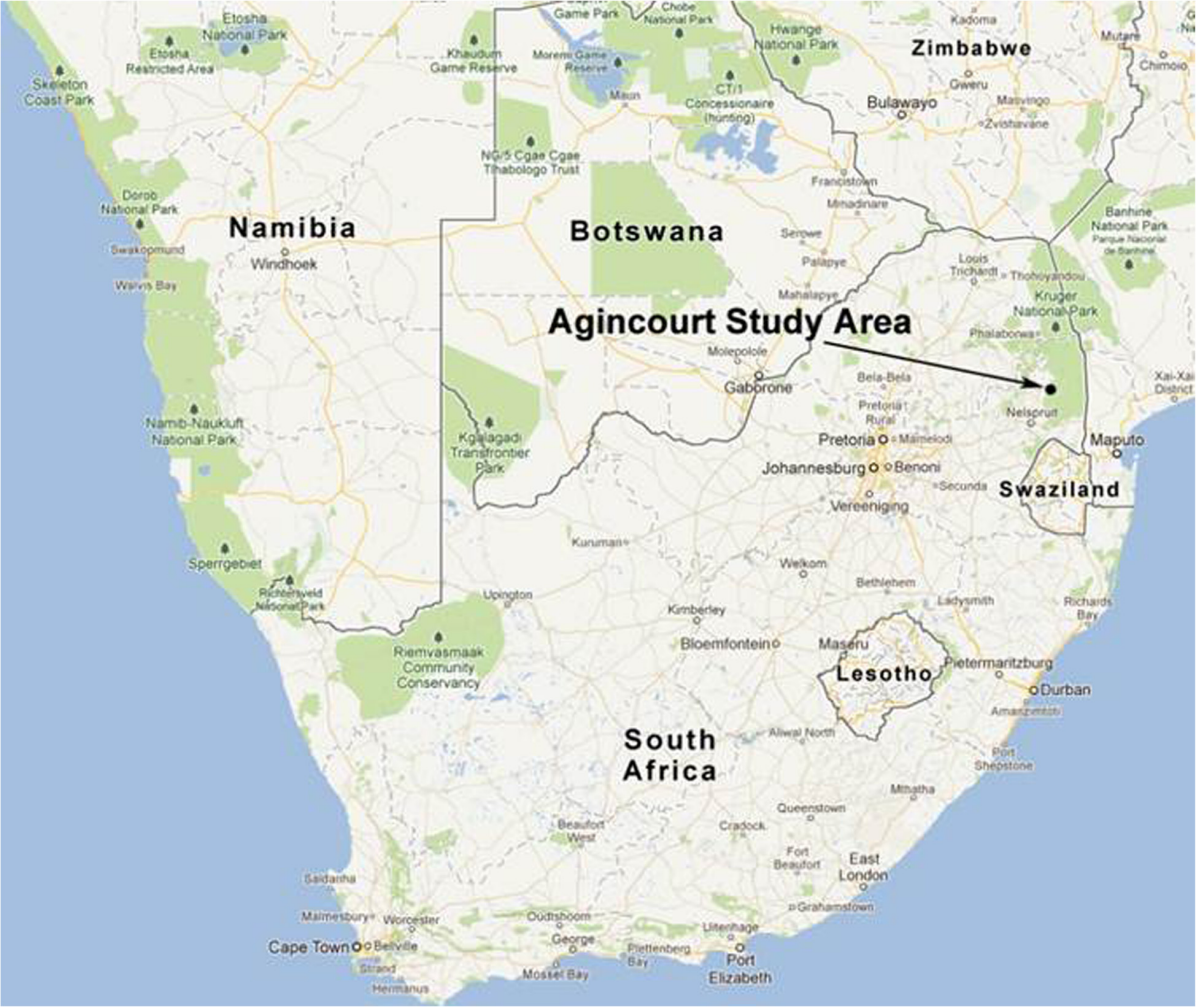
**The map of Agincourt HDSS, located in rural North-Eastern South Africa.** As of 2013, the surveillance site covered 32 villages and a population of more than 100,000 people.

This analysis is based on a population of approximately 70,000 people residing in the Agincourt sub-district of Mpumalanga province, north-eastern of South Africa between 2007 and 2011 (Figure 1) [19]. The area is completely covered by a health and demographic surveillance system (HDSS). Comprehensive data on mortality and causes of death, births, and inward and outward migration have been collected through a yearly census update since 1992. Additional data on labour participation and educational status have been collected at different time intervals to complement demographic data and provide contextual information. Agincourt sub-district has characteristics similar to many other rural South African populations. Though the sub-district’s socio-economic status has improved since 1994, the majority of the population relies on social assistance grants particularly pension and child care grants. Labour migration is high, with approximately 50-70% of men aged 20–59 years migrating to work outside the study area in 2011 [20]. The proportion is lower for women but increasing over the years with 25-35% of women considered a temporary migrant in 2011, an increase from 20-25% observed in 2000 [20].

The health status profile in the area is characterized by the persistent burden of TB and HIV/AIDS, maternal and child health problems, and emerging non-communicable diseases. Data from the HDSS suggest that between 1992 and 2005, cardiovascular disease (CVD) remained the top cause of death amongst women 50–64 years old [21]. In men, CVD showed a sustained increase. By 2002 it was the third-leading cause of death in men aged 50–64 and second-leading cause of death in men 65 years and above. The sub-district, measuring some 420 km^2^ is served by six clinics and one health centre. Hospital services are provided by three hospitals situated between 25 km and 45 km from the Agincourt study site. Imaging equipment for the diagnosis of stroke is lacking within the district but accessible at the provincial capital, Nelspruit, some 120 km south of Agincourt. At the time of this study, the stroke register set up in the early 2000s as part of the Southern African Stroke Prevention Initiative (SASPI) was no longer functional and there were no dedicated stroke units in the South African public health system.

### Ethics approval

Ethical approval for the study was granted by the Committee for Research on Human Subjects (Medical) of the University of the Witwatersrand, Johannesburg, South Africa for both the MRC/Wits Rural Public Health and Health Transitions Research Unit’s (Agincourt) Health and Socio-Demographic Surveillance System and add-on modules (Clearance certificate no. M131050).

### Computation of DALYs

DALYs were calculated by predominantly applying the methodological principles employed in the Global Burden of Disease Studies (detailed description in Additional file 1). DALYs are the sum of years of life lost due to premature mortality (YLL) plus years of life lost due to time lived in states of less than optimal health, loosely referred to as “disability” (YLD) [22]. YLL due to stroke among all persons that die of stroke is the sum of years that victims would have lived if they had completed the life expectancy attributed to their age (as assessed by a standard population) at the time of their death. In this study, the reference life table used in GBD 1990 study was chosen to ensure comparability with previous burden of disease studies. This was based on the highest life expectancy at the time, Japanese females with a life expectancy at birth of 82.5 years. YLLs measure the fatal burden of disease. The YLD figure expresses the consequences of living with less than perfect health conditions. It is an estimate based on the length of time that a condition persisted along with any accompanying disability and thus is an indicator of the non-fatal burden of disease. YLD can be calculated from an incidence perspective as the product of incidence, disability weights and average duration of disease. Alternatively YLD can be measured from a prevalence perspective as the product of prevalence of disease and disability weights.

To ensure consistency with the YLL calculation, which takes an inherently incidence perspective, and for comparison with earlier GBD studies, we compute incidence YLDs. Prevalence-based YLDs were calculated mainly for comparison with the GBD 2010 study. To be consistent with GBD 2010, we did not discount or apply age weighting in computing prevalence- based DALYs but apply discounting when YLDs are calculated using incidence. The latter allows comparison with earlier studies that discounted DALYs. Comparative analysis of the incident and prevalence YLDs is warranted as the two are not directly comparable. The incidence approach does not reflect the current prevalent burden of disabling sequelae for a condition for which incidence might have been substantially reduced. Secondly, in an incidence perspective, all YLDs for a condition are assigned to the age-groups at which the condition is incident, whereas in many cases for health policy-making, the ages at which the loss of health is experienced are of most interest.

### Data sources for calculating YLD

#### Prevalence of stroke

We conducted a systematic literature search of studies conducted in rural South Africa on prevalence of stroke. The search yielded one study; the Southern African Stroke Prevention Initiative (SASPI) study conducted in 2001 within the Agincourt population [6]. In that study, fieldworkers questioned each household informant, systematically reviewed every individual in the household using a previously validated questionnaire, and asked the following question: “Has (person) ever had weakness down one side of the body?” and “Has (person) ever had a stroke?” If either question was answered positively, a clinician/neurologist visited individuals aged >15 years to clinically assess the possible diagnosis of stroke by performing a detailed assessment of the patient. Clinical assessment of possible stroke victims was lowest amongst migrant males 25–44 years. To account for this non-contact, the investigators adjusted the stroke rates in each 10-year age stratum and assumed the same proportion of stroke survivors in employed men as among predominantly unemployed men. Migrant labourers were included in the local population denominator as they consider the sub-district home and return to seek health care when too ill to work [23]. Given the rising trend in risk factors, notably hypertension, and the potential impact of such changes in prevalence on YLD within the population, a sensitivity analysis was conducted around the point estimate, using a range of ±15%.

### Disability weights

Disability following a stroke spans a wide spectrum. Most contemporary stroke research has assessed disability using the modified rankin scales (MRS), a commonly used ordinal scale that measures disability or dependence in conducting activities of daily living in stroke victims [24]. In the GBD 2010 study, five sequelae of stroke were assessed and disability was ranked according to the lay definitions shown in Table 1 [25]. The disability weights were calculated based on personal interviews in Bangladesh, Indonesia, Peru, and Tanzania; telephone interviews in the USA; and an open access web-based survey. To identify the distribution of the severity of stroke within the Agincourt population, we used Hoffmann’s study (2000) carried out in KwaZulu Natal, South Africa [26]. In the study, patients were recruited into a stroke database from 1992–1998 and a retrospective analysis undertaken of all patients aged 15–40 to establish whether their disability resulted from stroke. Though disability was assessed based on the MRS, similar distribution of severity on the basis of the GBD sequela definition was assumed in this study. The slight difference between the definition of disability on MRS and categories in GBD 2010 was regarded as acceptable by a consensus of members of an international collaborative stroke expert group. A weighted disability weight (DW) was calculated by multiplying each disability weight by the proportion of the population it represented.

**Table 1:** **Disability weights for stroke at each disability level, South Africa**

**GBD 2010 stroke sequale**	**Definition of score**	**% of stroke population in SA (Hoffman et al. 2000** [26]**)**	**Disability weight associated with score (95% CI)**
0	No symptoms	2	0
1	long-term consequences, mild	40	0.021 (0.011-0.037)
2	long-term consequences, moderate	23	0.076 (0.050-0.110)
3	long-term consequences, moderate plus cognition problems	12	0.312 (0.363-0.705)
4	long-term consequences, severe	15	0.539 (0.363-0.705)
5	long-term consequences, severe plus cognition problems	8	0.567 (0.394-0.738)

Weighted average disability weight across all disability levels: 0.18.

### Derivation of incidence and duration through computer-based modelling

Dismod II was used to calculate incidence and duration of stroke-related disability. It models transitions from being healthy, to the incidence of a specific disease, to death from the disease under study or death from other causes. Given three input parameters such as remission, case fatality and prevalence, DisMod II can generate age-specific and sex-specific estimates of disease incidence. Because remission is defined as ‘cure’ in Dismod, no remission (i.e. improvement from the input condition) is possible when modelling stroke survivors. Consequently, prevalence (from SASPI study 2001), post-28 day relative risk of mortality, and a remission rate of zero were used to yield estimates of incidence and duration as outputs.

### Post 28-day stroke mortality

Due to the high risk of mortality in the first 28 days following a stroke, prevalence reflects only those who survive this period; there is thus a need to calculate mortality post-28 days. We could not identify South African specific studies that assessed post-28 day mortality amongst stroke survivors. The best available data chosen as input parameters was based on a prospective study conducted in a rural demographic surveillance site in Hai district, Tanzania between June 2003 and June 2006 [27]. The results of post-stroke case fatality relate to follow up until June 2009, which is at least 3 years of follow-up amongst the cases (Additional file 1: Table S6 and Table S7) [28]. To the best of the authors’ knowledge this is the first published data of post-stroke mortality in Sub-Saharan Africa, based on an incident population and that reports on long-term case fatality.

### Mortality rate at 28 days post-stroke (28-day case fatality)

Incidence calculated through Dismod reflects those who survive the high mortality period (first 28 days after stroke) since the prevalence of stroke that was used as input data also reflects those who survive the high mortality period. To show incidence of all cases (those who die within 28 days plus those who survive past 28 days), equation 1 is used to make an adjustment.

Equation 1:

$$ \begin{array}{l} Incidence\  of\  stroke\  amongst\  those\  who\  survive\  the\  first\ 28 days\ \left( calculated\  in\  Dismod\right)\\ {}\kern11em =\kern0.75em  All\  in cident\  case s*\ \left(1-28\  day\  case\  fatality\  rate\right)\end{array} $$

In South Africa, two hospital-based studies found case fatality rates of 33% and 34%, the weighted average of which is 33% [29,30]. Because the studies are hospital based and many people die before they reach facilities, we elected to use the mortality rates at 28 days post-stroke from the study described above conducted in Tanzania [28].

### Extrapolating incidence and disability to entire rural South Africa

Incidence and YLD of stroke in the whole of rural South Africa were extrapolated based on mortality rates and prevalence observed in Agincourt sub-district. The total population for rural South Africa was based on 2011 estimates by Statistics South Africa for the ‘mostly rural’ municipalities (Additional file 1: Table S2).

## Results

In 2007–11, 394 deaths were assigned to stroke in Agincourt HDSS, a crude stroke-related mortality rate of 114 per 100,000 person years (Additional file 1: Table S1). Of these, 30% were in males. When age-standardised to the WHO population [31], stroke mortality was 172 per 100,000 person years.

We estimate that 168 cases of stroke (72.7 males) occur every year in Agincourt sub-district (Table 2). This translates to a crude-incidence rate of 244 per 100,000 person years and age-adjusted rate of 349 per 100,000 person years. Table 3 shows the extrapolated incidence of stroke to the entire rural South Africa, a population of approximately 13 million people (Additional file 1: Table S2). It shows that at least 33,500 strokes occurred in 2011. This gives a crude incidence rate of 259 cases per 100,000 person years (age adjusted incidence of 347 per 100,000 person years). Of these, 20,800 were in females.

**Table 2:** **Yearly YLL, YLD and DALYs in Agincourt sub-district calculated using an incidence-based approach**

	**Age**	**Average yearly population**	**Incidence/100,000**	**Incidence (N)**	**Duration of disability (Years)**	**YLD/100,000**	**YLD (N)**	**YLL**	**DALYs**	**DALYs/100,000**
**Males**	0-4	11212.2	0.0	0.0	0.0	0.0	0.0	0.0	0.0	0.0
	5-14	8202.8	0.0	0.0	0.0	0.0	0.0	0.0	0.0	0.0
	15-29	5848.2	109.6	6.4	2.9	57.1	3.3	10.8	14.1	241.8
	30-44	3389.8	332.6	11.3	2.7	160.0	5.4	66.2	71.7	2113.9
	45-59	2019.8	780.8	15.8	3.5	489.6	9.9	94.5	104.4	5170.5
	60-69	1230.4	647.2	8.0	2.8	326.4	4.0	65.0	69.0	5610.2
	70-79	677.6	1394.2	9.4	1.8	453.5	3.1	41.9	45.0	6636.5
	80+	446.6	2283.9	10.2	1.1	464.7	2.1	21.5	23.6	5286.6
	**Total**	**33027.4**	**220.2**	**72.7**	**2.7**	**108.6**	**35.9**	**300.0**	**335.9**	**1017.0**
**Females**	0-4	11336.8	0.0	0.0	0.0	0.0	0.0	0.0	0.0	0
	5-14	8178.4	13.0	1.1	5.8	13.5	1.1	0.0	1.1	13.5
	15-29	5976.0	127.2	7.6	5.2	118.7	7.1	65.8	72.9	1219.3
	30-44	3897.8	274.1	10.7	4.3	214.5	8.4	87.1	95.4	2448.7
	45-59	2611.2	675.0	17.6	2.8	343.8	9.0	169.8	178.8	6847.1
	60-69	1582.6	1219.5	19.3	2.6	560.8	8.9	109.9	118.8	7505.2
	70-79	1115.4	1327.9	14.8	2.6	621.6	6.9	127.6	134.5	12061.3
	80+	1217.4	1312.0	16.0	2.1	485.5	5.9	116.8	122.7	10077.2
	**Total**	**35915.6**	**266.6**	**95.7**	**3.3**	**160.7**	**57.7**	**676.9**	**734.6**	**2045.5**

**Table 3:** **Incidence of stroke per 100,000 person years in 'mostly rural' South Africa, 2011 compared with national estimates**

**Males**				**Females**			**Both sexes, Agincourt**			**South Africa, National estimates 2008 (Bertram et al. 2013** [5]**)**	
**Age**	**Population**	**Incidence/100,000**	**Incidence (N)**	**Population**	**Incidence/100,000**	**Incidence (N)**	**Population**	**Incidence (N)**	**Incidence/100,000**	**Incidence/100,000 Males**	**Incidence/100,000 Females**
0-4	861642.0	0.0	0.0	850647.0	0.0	0.0	1712289.0	0.0	0.0	0	0
5-14	1569456.0	0.0	0.0	1515155.0	12.6	191.2	3084611.0	191.2	6.2	0	10
15-29	1832722.0	91.9	1685.2	1938675.0	121.6	2357.2	3771397.0	4042.4	107.2	79	143
30-44	789718.0	332.9	2628.7	1085763.0	278.0	3018.0	1875481.0	5646.7	301.1	412	423
45-59	532986.0	772.8	4118.9	824761.0	691.1	5699.7	1357747.0	9818.6	723.2	665	384
60-69	227238.0	645.8	1467.4	353604.0	1204.9	4260.7	580842.0	5728.2	986.2	583	609
70-79	119337.0	1401.3	1672.3	243683.0	1341.0	3267.8	363020.0	4940.1	1360.8	595	826
80+	53045.0	2252.2	1194.7	149895.0	1320.1	1978.7	202940.0	3173.4	1563.7	1384	2520
**Totals**	**5986144.0**	213.3 (198–243)*	**12767.2**	**6962183.0**	**298.4 (296–372)**	**20773.4**	**12948327.0**	**33540.6**	**259.0**	**465**	**615**

Comparison of stroke incidence rates in ‘mostly rural’ South Africa with national figures produced using similar modelling techniques shows that, incidence follows a similar pattern amongst males (Table 3). In females, incidence doubles from age group 30–44 to 45–59 in rural South Africa and follows a similar trend from age group 45–59 to 60–69. This is in contrast to the modest increases observed nationally amongst these age groups. The highest magnitude of increase amongst females at national level is observed between the last 2 age groups, where incidence increases from 826 to 2520 per 100,000 person years. However, results of uncertainty analysis (Additional file 1: Table S11 and Table S12) show that because the confidence intervals overlap from age 45–59 to highest age group there is no significant difference in incidence across these age groups.

### Burden of stroke in YLLs, YLDs and DALYs

Tables 2, 4, and 5 show the calculated YLLs, YLDs and DALYs lost in Agincourt sub-district for the period 2007–11. We present average yearly figures since burden of disease estimates are typically done for a year. In Tables 2 and 4, YLD estimates were produced using the incidence based approach with YLLs discounted to 3% as done in the global burden of disease study of 1990 and subsequent WHO updates. Table 5 presents DALYs calculated using the prevalence based approach, with no discounting (hereafter simplified DALY approach).

**Table 4:** **Yearly DALYs lost due to stroke in Agincourt sub-district, rural South Africa for both males and females based on incidence based approach**

**Age group**	**Population (% of total)**	**YLD**	**YLL**	**DALYs**	**DALYs/100,000**	**WHO standard population**	**Age-adjusted rates**
0-4	22549.0 (33%)	0.0	0.0	0.0	0.0	0.09	0.0
5-14	16381.2 (24%)	1.1	0.0	1.1	6.7	0.17	1.2
15-29	11824.2 (17%)	10.4	76.6	87.0	735.8	0.25	181.0
30-44	7287.6 (11%)	13.8	153.3	167.1	2293.0	0.21	489.5
45-59	4631.0 (7%)	18.9	264.4	283.2	6115.9	0.16	976.1
60-69	2813.0 (4%)	12.9	174.9	187.8	6676.3	0.07	446.0
70-79	1793.0 (3%)	10.0	169.5	179.5	10011.2	0.04	373.4
80+	1664.0 (2%)	8.0	138.3	146.3	8791.4	0.02	135.4
**Total**	**68943 (100%)**	**93.6**	**977.0**	**1070.5**	**1552.8**	**1.00**	**2602.6**

**Table 5:** **Yearly YLL, YLD, DALYs lost due to stroke in Agincourt sub-district, rural South Africa calculated using simplified DALY approach**

	**Age**	**Population**	**Prevalence**	**Disability weight**	**YLD /100,000**	**YLD (N)**	**YLL**	**DALYs**	**DALYs/100,000**
**Males**	0-4	11212.2	0.0	0.2	0.0	0.0	0.0	0.0	0.0
	5-14	8202.8	0.0	0.2	0.0	0.0	0.0	0.0	0.0
	15-29	5848.2	143.2	0.2	25.8	1.5	22.2	23.7	405.2
	30-44	3389.8	706.2	0.2	127.1	4.3	115.8	120.1	3543.1
	45-59	2019.8	1933.2	0.2	348.0	7.0	137.1	144.1	7134.6
	60-69	1230.4	2599.0	0.2	467.8	5.8	82.6	88.4	7183.5
	70-79	677.6	2854.2	0.2	513.7	3.5	49.1	52.6	7758.7
	80+	446.6	2839.7	0.2	511.1	2.3	23.1	25.3	5673.6
	**Totals**	**33027.4**	**471.9**	**0.2**	**84.9**	**28.1**	**429.8**	**457.9**	**1386.4**
**Females**	0-4	11336.8	0.0	0.2	0.0	0.0	0.0	0.0	0.0
	5-14	8178.4	17.1	0.2	3.1	0.3	0.0	0.3	3.1
	15-29	5976.0	319.9	0.2	57.6	3.4	138.6	142.1	2377.6
	30-44	3897.8	988.5	0.2	177.9	6.9	155.8	162.7	4174.9
	45-59	2611.2	1479.6	0.2	266.3	7.0	262.6	269.6	10324.5
	60-69	1582.6	2655.0	0.2	477.9	7.6	145.9	153.5	9698.4
	70-79	1115.4	3229.7	0.2	581.3	6.5	153.2	159.7	14317.8
	80+	1217.4	3208.1	0.2	577.5	7.0	128.5	135.5	11132.4
	**Totals**	**35915.6**	**680.6**	**0.2**	**122.5**	**44.0**	**984.7**	**1028.7**	**2864.2**
**Overall**	0-4	22549	0	0.2	0	0	0	0	0
	5-14	16381.2	17.1	0.2	1.5	0.3	0	0.3	1.5
	15-29	11824.2	463.1	0.2	41.9	4.9	160.8	165.8	1402
	30-44	7287.6	1694.7	0.2	154.3	11.2	271.6	282.8	3881
	45-59	4631	3412.8	0.2	301.9	14	399.7	413.7	8933.2
	60-69	2813	5254	0.2	473.5	13.3	228.6	241.9	8598.4
	70-79	1793	6083.9	0.2	555.8	10	202.3	212.3	11839
	80+	1664	6047.8	0.2	559.7	9.3	151.6	160.9	9667.3
	**Totals**	68943	1152.5	**0.2**	104.5	72.1	1414.5	1486.6	2156.3

We estimated that total DALYs lost due to stroke were 1,550 per 100,000 from an incidence-based perspective (Table 1). When the simplified DALY approach was used, DALYs lost due to stroke increased to 2,200 per 100,000 person years (Table 5). In both scenarios, YLDs comprised less than 10% of the DALYs lost. However, the ratio was much lower for prevalence-based YLD (3.3%) than for incidence-based (8.7%).

### Sensitivity analysis

Additional file 1: Table S8 shows the results of the sensitivity analysis. It highlights that case fatality rate has the most significant impact on overall crude incidence rate. Varying the 28 day case-fatality rate from 0.238 to 0.33 resulted in an increase in incidence rate of approximately 13.5%. 0.238 was the case-fatality rate reported by Walker and colleagues and used in this study, whilst 0.33 was the average case-fatality rate from 2 published hospital-based studies in South Africa [29,30]. Nonetheless neither case-fatality rate nor disability weight had a significant impact on overall DALYs lost. This is because both parameters predominantly affect YLD which in turn comprises a very small proportion of DALYs lost in Agincourt sub-district.

## Discussion

Emerging evidence suggests that rural South Africa is experiencing dramatic epidemiological changes characterised by an increase in non-communicable diseases (NCDs), persisting HIV/AIDS and TB and maternal and perinatal health issues [32]. During the period 1994 – 2009, NCD deaths increased amongst people under the age of 60 in Agincourt sub-district. At the same time, deaths due to HIV/AIDs initially decreased, then plateaued at high levels. To effectively address the colliding epidemics and intervene at a population level, empirical information about the scale of burden is required. However, there is a dearth of information, particularly incidence and morbidity of non-communicable diseases in rural South Africa.

This is the first study to calculate the burden of stroke in terms of both morbidity and mortality in rural populations of South Africa. The study begins the process of stratifying burden of the disease by urban and rural areas. Although the spectrum of urban to rural is clearly a continuum, a working approach to rural can support greater awareness and better targeted policy towards traditionally neglected populations with less functional health systems. Previous studies indicated that the burden of stroke might be lower in rural areas but the comparison was based on mortality and prevalence only and the data used is now out of date [5]. The major strength of this study is its use of predominantly local data from the Agincourt HDSS and from South Africa, with the exception of relative risk data.

Our findings indicate that the burden of stroke has increased over time in Agincourt sub-district. The observed stroke mortality rate of 114 per 100,000 person years in 2007–11 was significantly higher than the 87 per 100,000 person years observed in 1990–94 within the same population [8]. This trend towards an increased mortality rate is in contrast with what was reported in the GBD 2010 study for LMICs [2]. According to this study, mortality rates for ischaemic and haemorrhagic strokes declined between 1990 and 2010 in LMICs. Our findings could thus be reflective of the continual challenges that populations in rural South Africa face when accessing acute care services. Currently, imaging equipment for the diagnosis of stroke is accessible at the provincial capital, Nelspruit, some 120 km south of Agincourt and barriers to accessing care including the costs of transportation are well documented [33].

The burden of stroke seems to be disproportionately higher amongst rural populations in South Africa. According to Bertram et al. 2013 who used similar modelling techniques to measure incidence, approximately 75,000 strokes occur yearly in South Africa [5]. Our estimate of 33,500 strokes per year thus suggest that, despite comprising one-fifth of the total national population, “mostly rural” South Africa carries at least half of the stroke burden. However, it is important to mention that the national study was based on 2008 population estimates whilst our study was based on 2011 census estimates. We estimated a crude stroke rate of 244 per 100, 000 person years (CI: 110–122) within Agincourt sub-district. This is significantly higher than 108.6 per 100,000 person years reported for a Tanzanian rural demographic surveillance site [27]. Differences in incidence of stroke could indicate that rural SA is indeed in a health transition due to a higher level of lifestyle risk factor exposure [7]. However, other factors such as methodological differences in the 2 studies could explain the differences. When compared to global estimates from GBD 2010 study which ranged from 60 cases (Kuwait) to 504 cases (Lithuania) per 100 000 person-years [34]; our estimate falls within the middle of the range and these findings are consistent with the theory of an epidemiological transition occurring in rural South Africa.

We compared the age-adjusted incidence results for “mostly rural” South Africa with rural settings from middle –income countries for which data was available. The comparative analysis reveals that age-standardised incidence of stroke of 347 per 100,000 person years was higher than both West Bengal (262 per 100,000 person-years) and Trivandrum in rural India [35,36]. There are important differences between the Indian and rural South African settings that could account for the differences. In West Bengal, as in the rest of India, smoking is still a critical challenge and higher than optimal blood pressure was also evident amongst stroke survivors. It is estimated that smoking increases the odds of a stroke event by four. In South Africa, high blood pressure could be the most important underlying risk factor. According to the SASPI study, 43% of stroke survivors were hypertensive in Agincourt sub-district (BP greater than 140/90 mmHg). Though not examined in this study, the high HIV/AIDs prevalence in rural South Africa could account for some of the stroke cases. Emerging literature suggests that HIV infection can result in stroke via several mechanisms, including opportunistic infection, vasculopathy, cardioembolism, and coagulopathy [37]. HIV prevalence is highest in South Africa, with prevalence rates in Agincourt of 19.4% across all ages in 2010; peaking at 45.3% among men and at 46.1% among women, both at ages 35–39 [38]. This dual stroke/HIV burden reinforces the need to understand the underlying etiology of stroke in populations living with HIV/AIDS, particularly the younger age groups in which risk factors for stroke are seldom evident.

DALYs which have now been widely accepted by public health experts as suitable for measuring disease burden were also higher than most countries’ estimates. According to the GBD study, in 2010, DALYs lost due to stroke ranged from 398 (Australia) to 5227 (Afghanistan) per 100 000 people [34]. In LMICs, DALY loss rate per 100,000 person years amongst all ages was 1821 (1589–1925), and this was twice as high as the estimates produced for high income countries. During that period, DALY loss rate per 100,000 person years for the whole of South Africa was estimated to be 1,570 (1,381.20-1,926.24). This places our estimated DALY loss rate in rural Agincourt of 1,552 per 100,000 person years within the upper range and suggests a significant burden due to stroke.

Comparing the DALYs computed based on the GBD 2010 methodology where time discount of 0% and no age-weighting were applied, we observed a substantial increase in the absolute number of DALYs lost (Table 5). Time discounting places less importance on health benefits that occur in the future and as such its removal will result in the observed increases in DALYs lost, particularly if the stroke death occurred amongst younger age groups. These findings are in line with what was reported in a recent WHO analysis [39]. These differences in methodological approaches warrant caution when conducting cross-country comparisons of DALY results and reinforce the need to explain in depth the methodological approach taken when calculating DALYs.

The high fatal burden of stroke warrants urgent measures for population-wide stroke interventions. Potential interventions include lowering sodium content of processed foods. This intervention could prevent 7 400 CVD deaths and 4 300 non-fatal strokes per year in South Africa and initiatives are underway to implement this policy [40]. In a setting where 30% of people with diagnosed hypertension are on correct medication and have controlled blood pressure [41], personal interventions are equally important. Such approaches could include pharmacological treatment of individuals with global risk of CVD [42]. There are other novel initiatives that are currently being explored in rural SA. The Nkateko (Hope) trial is one such example [43]. This cluster randomised trial, focused on integrated chronic care will evaluate clinic-based lay health worker support for community health worker efforts to manage NCDs, hypertension in particular. . Future analyses should thus focus on estimating the cost-effectiveness of strategies to reduce the stroke burden. This study provides a platform through which such analyses can be made.

Despite the strengths of our analysis, there are a number of limitations in the data used. The data did not distinguish between haemorrhagic and ischemic stroke. This requires radiological imaging which is costly and not readily accessible. The inability to distinguish between stroke subtypes makes it difficult to accurately determine the appropriate epidemiological parameters to use particularly regarding case fatality. Different pathological subtypes result in different prognoses and knowledge of the pathological type of stroke is important for targeted country-specific health-care planning.

Cause of death was ascertained through verbal autopsy in Agincourt HDSS, not physician coding [19]. Verbal autopsy would tend to misclassify deaths. However, we do not believe that this omission had a significant impact on the results of this analysis because verbal autopsy methods for assigning stroke deaths have been well validated, particularly for assigning fatal stroke deaths [44].

## Conclusions

By utilizing population-based data from a well characterised research setting, coupled with modelling techniques, we were able to derive the first estimate of total burden of stroke in a rural South African population. The findings provide further evidence on the dynamic health transition underway, and suggest that stroke is on the increase in rural areas of South Africa despite the immense impact of HIV/AIDS. In the absence of health system interventions, rural South Africa appears at risk for a stroke epidemic. Furthermore, the study highlights the critical role played by indepth studies in understanding the burden of disease in countries where health information systems are not adequate. Agincourt’s longitudinal research platform can monitor the evolution of stroke mortality in rural South Africa, particularly the impact of targeted interventions. This study is well positioned to advance the health policy agenda of South Africa, in particular as outlined in the ‘South African Declaration on the Prevention and Control of Non-communicable Diseases’ adopted in 2011. One of the key objectives of that strategic plan was to develop an integrated and inter-sectoral plan for a coordinated response to prevention of non-communicable diseases. While noble, this goal cannot be accomplished without proper baseline data and information on high burden non-communicable diseases.

### Recommendations

Future research in South Africa should focus on obtaining population representative data on stroke incidence to strengthen burden-of-disease analysis. Such studies will allow derivation of missing epidemiological parameters, particularly incidence and case fatality, and will enable a fuller understanding of stroke epidemiology in rural areas. It would be worthwhile to conduct such studies within health and demographic surveillance sites in which mortality data and/or prevalence data can be readily sourced. In addition, a follow-up study to SASPI would enable South Africa to track the extent to which prevalence of stroke has increased in rural areas. Data on disability is very difficult to obtain with completeness and reliability and stroke registers are vital.

## Additional file

Additional file 1:
**Supplementary appendix with detailed methodology including population figures, data sources and results of uncertainty analysis.**

## Abbreviations

CVD: Cardiovascular diseases
DALY: Disability adjusted life year
DW: Disability weight
GBD: Global burden of disease
GDP: Gross domestic product
HDL: High density lipoprotein
HDSS: Health and socio-demographic surveillance system
IPM: Incidence-prevalence-mortality
LMICs: Low- and middle-income countries
MRS: Modified ranking scales
PDG: Palmer development group
PURE: Prospective urban rural epidemiology
SA: South Africa
SA NBD: South African national burden of disease
SASPI: Southern African stroke prevention initiative
SSBs: Sugar sweetened beverages
TB: Tuberculosis
YLD: Years lived with disability
YLL: Years of life lost
