# Supplementary material for: Disease burden of stroke in rural South Africa: an estimate of incidence, mortality and disability adjusted life years
Source: BMC Neurol. 2015 Apr 12;15:54. doi: 10.1186/s12883-015-0311-7 (PMC4396076; doi:10.1186/s12883-015-0311-7)
Supplement: Additional file 1: — Supplementary appendix with detailed methodology including population figures, data sources and results of uncertainty analysis. [file 12883_2015_311_MOESM1_ESM.docx]

**Disease burden of stroke in rural South Africa: an estimate of incidence, mortality and disability adjusted life years**

**Supplementary Appendix**

Mandy Maredza^1§^, Melanie Y Bertram^2^, Stephen M Tollman^1, 3, 4^

^1^MRC/Wits Rural Public Health and Health Transitions Research Unit (Agincourt), School of Public Health, Faculty of Health Sciences, University of the Witwatersrand, Johannesburg, South Africa

^2^ World Health Organization, Geneva, Switzerland

^3^Centre for Global Health Research, Umeå University, Sweden

^4^INDEPTH Network, Accra, Ghana

^§^ Corresponding author

MRC/Wits Rural Public Health and Health Transitions Research Unit (Agincourt), School of Public Health

University of the Witwatersrand

Education Campus

St Andrews Road

Parktown,

Johannesburg, South Africa

Tel: +27 21 483 2677

Cell: +27 712 640 918

Email addresses:

MM: [rmtanya@gmail.com](mailto:rmtanya@gmail.com)

MYB: [bertramm@who.int](mailto:bertramm@who.int)

SMT: [Stephen.Tollman@wits.ac.za](mailto:Stephen.Tollman@wits.ac.za)

1. Population figures for Agincourt HDSS and 70 ‘mostly rural’ municipalities

Table 1: Total number of deaths and population in Agincourt 2007-2011

|  |  |  |  |  |  |  |  |  |  |  |
| --- | --- | --- | --- | --- | --- | --- | --- | --- | --- | --- |
|  | **Person-years (over 5 yrs)** | | **Deaths from any cause** | | **Mortality rate** | | **Population numbers** | | **Deaths due to stroke** | |
|  | **Males** | **Females** | **Males** | **Females** | **Male** | **Female** | **Male** | **Female** | **Male** | **Female** |
| 0 | 2 767 | 2 751 | 68 | 44 | 0.025 | 0.016 | 553 | 550 | 0 | 0 |
| 1-4 | 15 624 | 15 723 | 137 | 104 | 0.009 | 0.007 | 3125 | 3145 | 0 | 0 |
| 5-9 | 18 649 | 18 840 | 52 | 39 | 0.003 | 0.002 | 3730 | 3768 | 0 | 0 |
| 10-14 | 19 021 | 19 370 | 72 | 42 | 0.004 | 0.002 | 3804 | 3874 | 0 | 0 |
| 15-19 | 20 445 | 20 591 | 41 | 95 | 0.002 | 0.005 | 4089 | 4118 | 0 | 2 |
| 20-24 | 20 569 | 20 301 | 134 | 176 | 0.007 | 0.009 | 4114 | 4060 | 1 | 1 |
| 25-29 | 16 417 | 16 736 | 276 | 454 | 0.017 | 0.027 | 3283 | 3347 | 1 | 9 |
| 30-34 | 12 824 | 13 144 | 432 | 446 | 0.034 | 0.034 | 2565 | 2629 | 1 | 2 |
| 35-39 | 9 440 | 10 479 | 346 | 340 | 0.037 | 0.032 | 1888 | 2096 | 7 | 4 |
| 40-44 | 7 509 | 9 010 | 492 | 275 | 0.066 | 0.031 | 1502 | 1802 | 6 | 12 |
| 45-49 | 5 770 | 7 332 | 308 | 252 | 0.053 | 0.034 | 1154 | 1466 | 0 | 10 |
| 50-54 | 4 329 | 5 724 | 309 | 233 | 0.071 | 0.041 | 866 | 1145 | 13 | 18 |
| 55-59 | 3 626 | 4 548 | 238 | 201 | 0.066 | 0.044 | 725 | 910 | 13 | 14 |
| 60-64 | 2 526 | 3 365 | 231 | 205 | 0.091 | 0.061 | 505 | 673 | 6 | 12 |
| 65-69 | 1 751 | 2 984 | 235 | 204 | 0.134 | 0.068 | 350 | 597 | 19 | 25 |
| 70-74 | 1 637 | 2 593 | 200 | 205 | 0.122 | 0.079 | 327 | 519 | 14 | 30 |
| 75-79 | 795 | 2 325 | 151 | 213 | 0.190 | 0.092 | 159 | 465 | 9 | 32 |
| 80-84 | 713 | 2 189 | 174 | 295 | 0.244 | 0.135 | 143 | 438 | 7 | 58 |
| 85-89 | 725 | 1 573 | 221 | 281 | 0.305 | 0.179 | 145 | 315 | 20 | 48 |
| Total | 165 137 | 179 578 | 4 117 | 4 104 | 0.025 | 0.023 | 33 027 | 35 916 | 117 | 277 |

Table 2: Total population figures by age and sex in 70 “mostly rural” municipalities of South Africa (Statistics South Africa, 2011)

| Age Group | **Male** | **Female** | **Total** |
| --- | --- | --- | --- |
| 0 - 4 | 861 642 | 850 647 | 1 712 289 |
| 5 - 9 | 781 287 | 771 497 | 1 552 784 |
| 10 - 14 | 788 169 | 743 658 | 1 531 827 |
| 15 - 19 | 814 390 | 792 860 | 1 607 250 |
| 20 - 24 | 593 558 | 630 462 | 1 224 020 |
| 25 - 29 | 424 774 | 515 353 | 940 127 |
| 30 - 34 | 312 116 | 401 640 | 713 756 |
| 35 - 39 | 262 005 | 360 947 | 622 952 |
| 40 - 44 | 215 597 | 323 176 | 538 773 |
| 45 - 49 | 199 145 | 316 641 | 515 786 |
| 50 - 54 | 176 989 | 274 667 | 451 656 |
| 55 - 59 | 156 852 | 233 453 | 390 305 |
| 60 - 64 | 134 876 | 200 053 | 334 929 |
| 65 - 69 | 92 362 | 153 551 | 245 913 |
| 70 - 74 | 77 401 | 138 986 | 216 387 |
| 75 - 79 | 41 936 | 104 697 | 146 633 |
| 80 - 84 | 29 739 | 83 493 | 113 232 |
| 85+ | 23 306 | 66 402 | 89 708 |
| **Total** | 5 986 137 | 6 962 183 | 12 948 320 |

Table 3: Classification of municipalities in South Africa (National Treasury, 2011)

| Class | Characteristics | Municipalities country-wide (N) |
| --- | --- | --- |
| Metros | Category A municipalities | 6 |
| Secondary Cities | All local municipalities referred to as secondary cities | 21 |
| Large Towns | All local municipalities with an urban core. There is huge variation in population sizes amongst these municipalities and they do have large urban dwelling population. | 29 |
| Small Towns | They are characterised by no large town as a core urban settlement. Typically, these municipalities have a relatively small population, a significant proportion of which is urban and based in one or more small towns. Rural areas in this category are characterised by the presence of commercial farms, as these local economies are largely agriculturally based. The existence of such important rural areas and agriculture sector explains their inclusion in the analysis of rural municipalities. | 111 |
| Mostly Rural | These are characterised by the presence of at most one or two small towns in their areas, communal land tenure and villages or scattered groups of dwellings and typically located in former homelands | 70 |
| Districts | District municipalities that are not water services providers. | 25 |
| Districts | District municipalities that are water service providers | 21 |

1. **Computation of DALYs**

DALYs were calculated by predominantly applying the methodological principles employed in the Global Burden of Disease Studies. DALYs are the sum of years of life lost due to premature mortality (YLL) plus years of life lost due to time lived in states of less than optimal health, loosely referred to as “disability” (YLD) (equation 1) [[28](#_ENREF_28)]. YLL due to stroke among all persons that die of stroke is the sum of years that victims would have lived if they had completed the life expectancy attributed to their age (as assessed by a standard population) at the time of their death. YLLs measure the fatal burden of disease. The YLD figure expresses the consequences of living with less than perfect health conditions. It is an estimate based on the length of time that a condition persisted along with any accompanying disability and thus is an indicator of the non-fatal burden of disease. One DALY can be thought of as one lost year of ‘healthy’ life whilst the measured disease burden is the gap between a population’s health status and that of a normative reference population [[29](#_ENREF_29)].

Thus,

$${DALY}_{i}={YLL}_{i}+{YLD}_{i}$$

*Estimating fatal burden of disease*

The basic formula for calculating YLL for a particular cause, age or sex is

YLL = N × L,

where N is the number of deaths and L is the standard life expectancy (in years) at the age of death. The standard reference life-table is intended to represent the potential maximum life span of an individual in good health at a given age. We chose the reference life tables used in GBD 1990 study which was based on the highest life expectancy at the time, Japanese females with a life expectancy at birth of 82.5 years, to ensure comparability with previous burden of disease studies. However, YLLs based on the GBD 2010 study reference life table were also calculated to allow comparison with the GBD 2010 study. The life table for GBD 2010 study was based on the lowest observed death rate for each age group in countries with populations of more than 5 million, and the new life expectancy at birth was set at 86 years.

.

*Estimating non-fatal burden of disease*

The basic formula for calculating YLD for a particular disability event is

YLD = I × DW × L,

where:

I is the number of incident cases in period; DW is the disability weight and reflects the severity of the disease on a scale from 0 (perfect health) to 1 (dead); L is the average duration of disease (in years).

The recent GBD 2010 study based the YLD calculation on prevalence rather than incidence:

$$YLD=P x DW$$

where:

P = number of prevalent cases

DW = disability weight

To ensure consistency with the YLL calculation, which takes an inherently incidence perspective, and for comparison with earlier GBD studies, we compute incidence YLDs. Prevalence-based YLDs were calculated mainly for comparison with the GBD 2010 study.. To be consistent with GBD 2010, we did not discount or apply age weighting in computing prevalence- based DALYs but apply discounting when YLDs are calculated using incidence.. The latter allows comparison with earlier studies that discounted DALYs. Comparative analysis of the incident and prevalence YLDs is warranted as the two are not directly comparable. The incidence approach does not reflect the current prevalent burden of disabling sequelae for a condition for which incidence might have been substantially reduced. Secondly, in an incidence perspective, all YLDs for a condition are assigned to the age-groups at which the condition is incident, whereas in many cases for health policy-making, the ages at which the loss of health is experienced are of most interest

1. **Computation of YLLs due to stroke**

Table 4: YLLs due to stroke calculated based on reference population

|  | Standard population | |  |  |  | Derived life expectancy at Agincourt   (undiscounted) | | Discounted life expectancy | |  | YLL Undiscounted | | YLL discounted | |
| --- | --- | --- | --- | --- | --- | --- | --- | --- | --- | --- | --- | --- | --- | --- |
| Age | Males | Females |  | Age group | Age at death  In Agincourt | Males | Females | Males | Females |  | Males | Females | Males | Females |
| **A** | **B** | **C** | **D** | **E** | **F** | **G** | **H** | **I** | **J** | **K** | **L** | **M** | **N** | **O** |
| 0 | 80.00 | 82.50 |  | 0 | 0.05 | 79.97 | 82.47 | 30.31 | 30.53 |  | 0.00 | 0.00 | 0.00 | 0.00 |
| 1 | 79.36 | 81.84 |  | 1-4 | 1.65 | 78.72 | 81.21 | 30.19 | 30.42 |  | 0.00 | 0.00 | 0.00 | 0.00 |
| 2 | 78.36 | 80.87 | 5.00 | 5-9 | 7.50 | 72.89 | 75.47 | 29.59 | 29.87 |  | 0.00 | 0.00 | 0.00 | 0.00 |
| 3 | 77.37 | 79.90 | 10.00 | 10-14 | 12.50 | 67.91 | 70.51 | 28.99 | 29.31 |  | 0.00 | 0.00 | 0.00 | 0.00 |
| 4 | 76.38 | 78.92 | 15.00 | 15-19 | 17.50 | 62.93 | 65.55 | 28.29 | 28.67 |  | 0.00 | 131.10 | 0.00 | 57.34 |
| 5 | 75.38 | 77.95 | 20.00 | 20-24 | 22.50 | 57.95 | 60.63 | 27.47 | 27.93 |  | 57.95 | 60.63 | 27.47 | 27.93 |
| 6 | 74.39 | 76.96 | 25.00 | 25-29 | 27.50 | 52.99 | 55.72 | 26.53 | 27.07 |  | 52.99 | 501.48 | 26.53 | 243.62 |
| 7 | 73.39 | 75.97 | 30.00 | 30-34 | 32.50 | 48.04 | 50.83 | 25.44 | 26.08 |  | 48.04 | 101.65 | 25.44 | 52.15 |
| 8 | 72.39 | 74.97 | 35.00 | 35-39 | 37.50 | 43.10 | 45.96 | 24.19 | 24.94 |  | 301.72 | 183.82 | 169.30 | 99.74 |
| 9 | 71.40 | 73.98 | 40.00 | 40-44 | 42.50 | 38.20 | 41.13 | 22.74 | 23.63 |  | 229.22 | 493.50 | 136.43 | 283.52 |

Table 5: Calculations for YLLs as they relate to table above

|  | Standard population | |  |  |  | Derived life expectancy at Agincourt (undiscounted) | | Discounted life expectancy | |  | YLL Undiscounted | | YLL discounted | |
| --- | --- | --- | --- | --- | --- | --- | --- | --- | --- | --- | --- | --- | --- | --- |
| Age | Males | Females |  | Age group | Average at death in Agincourt | Males | Females | Males | Females |  | Males | Females | Males | Females |
| **A** | **B** | **C** | **D** | **E** | **F** | **G** | **H** | **I** | **J** | **K** | **L** | **M** | **N** | **O** |
| 0 | 80 | 82.5 |  | 0 | 0.045 | =B8+F8*(B9-B8) | =C8+F8*(C9-C8) | =(1-EXP(-discount rate*G8))/ discount rate | =(1-EXP(-discount rate*H8))/discount rate |  | =G8*# of stroke deaths at this age | =H8*# of stroke deaths at this age | =I8*# of stroke deaths at this age | =J8**# of stroke deaths at this age |
| 1 | 79.358 | 81.84 |  | 1-4 | 1.645 | =B10+($F9-$A10)*(B11-B10) | =C10+($F9-$A10)*(C11-C10) | =(1-EXP(-discount rate *G9))/ discount rate | =(1-EXP(-discount rate*H9))/discount rate |  | =G9*# of stroke deaths at this age | =H9*# of stroke deaths at this age | =I9*# stroke deaths at this age | =J9*# stroke deaths this age |

Table 6: Age and sex specific cause death rate at 28days post-stroke (Walker et al 2011)

| geAge^[*](http://0-jnnp.bmj.com.innopac.wits.ac.za/content/82/9/1001/T2.expansion.html#fn-2)^ | Cases identified by TSIP | | Cases identified by VA | |
| --- | --- | --- | --- | --- |
|  | Males | Females | Males | Females |
|  | No of deaths/total cases (%) | No of deaths (%) | No of deaths (%) | No of deaths (%) |
| 0–44 years | 0/3 (0%) | 1/6 (16.7%) | 2/4 (50.0%) | 3/13 (23.1%) |
| 45–54 years | 2/6 (33.3%) | 0/5 (0%) | 2/8 (25.5%) | 4/11 (36.4%) |
| 55–64 years | 4/10 (40.0%) | 3/11 (27.3%) | 2/14 (14.3%) | 2/11 (18.2%) |
| 65–74 years | 3/22 (13.6%) | 5/18 (27.8%) | 5/35 (14.3%) | 8/24 (33.3%) |
| 75–84 years | 5/21 (23.8%) | 3/8 (37.5%) | 15/42 (35.7%) | 9/32 (28.1%) |
| ≥85 years | 1/7 (14.3%) | 4/13 (30.8%) | 5/11 (45.5%) | 7/18 (38.9%) |
| Overall | 15/69 (21.7%) | 16/61 (26.2%) | 31/114 (27.2%) | 33/109 (30.3%) |
| Mean age (years) at stroke of those dead | 70.5 (95% CI 65.8 to 75.2) | | 71.3 (95% CI 67.0 to 75.6) | |
| Mean age (years) at stroke of those alive | 68.2 (95% CI 65.1 to 71.2) | | 69.7 (95% CI 67.5 to 72.0) | |

- [↵](http://0-jnnp.bmj.com.innopac.wits.ac.za/content/82/9/1001/T2.expansion.html#xref-fn-2-1)***** Ages given are at time of stroke.

Table 7: Age and sex-specific cause death rates at 3years post stroke (Walker et al 2011)

| Age[^*^](http://0-jnnp.bmj.com.innopac.wits.ac.za/content/82/9/1001/T3.expansion.html#fn-4) | Cases identified by TSIP | | Cases identified by VA | |
| --- | --- | --- | --- | --- |
|  | Males | Females | Males | Females |
|  | No of deaths/total cases (%) | No of deaths (%) | No of deaths (%) | No of deaths (%) |
| 0–44 years | 1/3 (33.3%) | 2/6 (33.3%) | 4/4 (100.0%) | 8/13 (61.5%) |
| 45–54 years | 4/6 (66.7%) | 2/5 (40.0%) | 6/8 (75.5%) | 7/11 (63.6%) |
| 55–64 years | 5/10 (50.0%) | 6/11 (54.5%) | 11/14 (78.6%) | 8/11 (72.7%) |
| 65–74 years | 10/22 (45.5%) | 12/18 (66.7%) | 29/35 (82.9%) | 20/24 (83.3%) |
| 75–84 years | 16/21 (76.2%) | 5/8 (62.5%) | 40/42 (95.2%) | 30/32 (93.8%) |
| ≥85 years | 6/7 (85.7%) | 9/13 (69.2%) | 9/11 (81.8%) | 16/18 (88.9%) |
| Overall | 42/69 (60.9%) | 36/61 (26.2%) | 99/114 (86.8%) | 89/109 (81.7%) |
| Mean age (years) at stroke of those dead | 71.2 (95% CI 68.0 to 74.3) | | 71.6 (95% CI 69.5 to 73.7) | |
| Mean age (years) at stroke of those alive | 65.0 (95% CI 60.7 to 69.4) | | 62.5 (95% CI 57.1 to 67.9) | |

Table 8: Sensitivity analysis of changes in DALYs due to change in case fatality and disability weight input parameters

|  |  |  | **Incidence-based DALY** | | **Prevalence-based DALY** |
| --- | --- | --- | --- | --- | --- |
| **Parameter** | **Value** | **Source** | **Incidence/100,000** | **DALY/100,000** | **DALY/100,000** |
| 28-day CFR | 0.238 | Walker et al 2011 | 244 | 1552 | N/A |
|  | 0.33 |  | 277* | 1571.4 | N/A |
| Disability weight | 0.18 | Calculated in this study | 244 | 1552 | 2156 |
|  | 0.25 | Bertram et al | 244 | 1605 | 2196 |
| Combined DW + CFR | 0.238; 0.18 |  | 244 | 1552 | 2156 |
|  | 0.33; 0.25 |  | 277 | 1631 | 2196 |
| *There is than a 10% difference in incidence when 28day CFR is changed from 0.238 to 0.33. However, changes in CFR or DW do not significantly affect DALY results. | | | | | |

Table 9: Dismod output for males in rural South Africa

| DisMod II input and output, database Example | | | | |  |  |  |  |  |  |  |
| --- | --- | --- | --- | --- | --- | --- | --- | --- | --- | --- | --- |
| Males, Disease: Stroke (Rates * 100,000), sex: Males | | | | | |  |  |  |  |  |  |
| Written 2015/01/22, 12:00:02 PM | | | |  |  |  |  |  |  |  |  |
| Age | Prevalence (rates * 100000) | Remission (rates * 100000) | Case fatality (rates * 100000) | Incidence (rates * 100000) | Prevalence (rates * 100000) | Remission (rates * 100000) | Case fatality (rates * 100000) | Duration (years) | Mortality (rates * 100000) | RR mortality (number) | Age of onset (years) |
| 0-4 | 0 | 0 | 33333.33 | 0 | 0 | 0 | 33333.33 | 0 | 0 | 0 | 2.3888 |
| 5-9 | 0 | 0 | 33333.33 | 0 | 0 | 0 | 33333.33 | 0 | 0 | 97.9448 | 8.5115 |
| 15-29 | 200.2512 | 0 | 33333.33 | 70.0654 | 118.7767 | 0.4684 | 32216.16 | 2.8943 | 38.5412 | 23.714 | 26.3361 |
| 30-44 | 714.6205 | 0 | 33333.33 | 253.6468 | 707.3427 | 0.6142 | 33145.36 | 2.6683 | 234.6233 | 9.2776 | 37.121 |
| 45-59 | 2469 | 0 | 25555.56 | 588.8698 | 1986.206 | 0.6142 | 23832.01 | 3.5375 | 474.3607 | 5.2936 | 51.3497 |
| 60-69 | 2619.795 | 0 | 20909.09 | 492.0761 | 2598.319 | 0.6142 | 17488.74 | 2.8047 | 454.5316 | 2.6782 | 65.6919 |
| 70-79 | 2840 | 0 | 42099.57 | 1067.789 | 2853.771 | 0.6142 | 37390.71 | 1.7955 | 1067.03 | 3.5411 | 74.5778 |
| 80+ | 2840 | 0 | 65476.19 | 1716.161 | 2839.655 | 0.6142 | 60420.15 | 1.1446 | 1715.69 | 3.3258 | 87.1851 |
| All ages | 556.6484 | NA | NA | 162.5183 | 487.2139 | 0.6033 | 28430.3 | 2.7373 | 138.7172 | 6.9397 | 53.1563 |

Incidence, and duration of disability calculated in Dismod are used for YLD calculations. However, incidence calculated in Dismod reflects those who survive the high mortality period (28days post stroke). To show incidence of all cases (those who die within 28days plus those who survive past 28days), we use equation 1 to make an adjustment. As such the figures shown in this table, will differ from what is indicated in table 4 (main manuscript)

Equation 1:

$$Incidence of stroke amongst those who survive the first 28days \left( calculated in Dismod \right)= All incident cases* (1-28 day case fatality rate)$$

Table 10: Dismod output for all females, rural South Africa (2011)

| DisMod II input and output, database Example | | | | |  |  |  |  |  |  |  |
| --- | --- | --- | --- | --- | --- | --- | --- | --- | --- | --- | --- |
| Females, Disease: Stroke (Rates * 100,000), sex: Females | | | | | |  |  |  |  |  |  |
| Written 2015/01/22, 11:58:53 AM | | | |  |  |  |  |  |  |  |  |
| Age | Prevalence (rates * 100000) | Remission (rates * 100000) | Case fatality (rates * 100000) | Incidence (rates * 100000) | Prevalence (rates * 100000) | Remission (rates * 100000) | Case fatality (rates * 100000) | Duration (years) | Mortality (rates * 100000) | RR mortality (number) | Age of onset (years) |
| 0-4 | 0 | 0 | 16666.67 | 0 | 0 | 0 | 0 | 0 | 0 | 0 | 2.5 |
| 5-9 | 0 | 0 | 16666.67 | 9.6158 | 16.5658 | 0.3574 | 16495.77 | 5.7878 | 2.7483 | 74.2581 | 12.7161 |
| 15-29 | 378.3188 | 0 | 16666.67 | 92.651 | 303.9778 | 0.6106 | 16664.85 | 5.1995 | 50.7549 | 15.3184 | 24.015 |
| 30-44 | 1052.262 | 0 | 16666.67 | 211.8057 | 1000.436 | 0.6142 | 17547.49 | 4.3222 | 175.6392 | 6.4945 | 37.9858 |
| 45-59 | 1549.475 | 0 | 35757.58 | 526.5974 | 1499.517 | 0.6142 | 30779.99 | 2.8313 | 462.1387 | 8.7696 | 53.4427 |
| 60-69 | 2721.188 | 0 | 33080.81 | 918.1675 | 2641.668 | 0.6142 | 31819.04 | 2.5635 | 841.2487 | 5.9659 | 65.1759 |
| 70-79 | 3210 | 0 | 31944.44 | 1021.857 | 3230.551 | 0.6142 | 31294.86 | 2.5985 | 1011.129 | 4.8251 | 74.2858 |
| 80+ | 3210 | 0 | 34254.81 | 1005.893 | 3208.253 | 0.6142 | 31275.37 | 2.0428 | 1003.4 | 3.0002 | 87.9055 |
| All ages | 772.6741 | NA | NA | 227.3615 | 738.2209 | 0.6125 | 26609.21 | 3.1772 | 196.5887 | 7.1692 | 56.5695 |

Table 11: Results of uncertainty analysis for incidence

| DisMod II input and output, database Example | | | | |  |  |  |  |  |  |  |
| --- | --- | --- | --- | --- | --- | --- | --- | --- | --- | --- | --- |
| Uncertainty results, Stroke Incidence (Rates * 100,000), sex: Males | | | | | | |  |  |  |  |  |
| Written 2015/01/25, 01:25:46 PM | | | |  |  |  |  |  |  |  |  |
| Age | Central value (rates * 100000) | Lower (rates * 100000) | Upper (rates * 100000) | Lower (rates * 100000) | Upper (rates * 100000) | Lower (rates * 100000) | Upper (rates * 100000) | Lower (rates * 100000) | Upper (rates * 100000) | Lower (rates * 100000) | Upper (rates * 100000) |
| 0-4 | 0 | 0 | 0 | 0 | 0 | 0 | 0 | 0 | 0 | Lower 95% | Upper 95% |
| 5-14 | 0.0005 | 0.0005 | 0.0005 | 0.0005 | 0.0005 | 0.0005 | 0.0005 | 0.0005 | 0.0005 | 0.0005 | 0.0005 |
| 15-29 | 82.7721 | 80.9025 | 84.65 | 80.3099 | 85.7169 | 79.6331 | 86.0738 | 78.9537 | 86.5566 | 77.6854 | 87.1297 |
| 30-44 | 362.7304 | 343.9262 | 381.7513 | 339.3563 | 386.4783 | 334.7465 | 393.5493 | 326.0037 | 400.8414 | 314.8333 | 402.0014 |
| 45-59 | 996.259 | 952.2208 | 1044.394 | 942.7682 | 1054.61 | 931.4384 | 1067.277 | 917.761 | 1082.992 | 895.63 | 1088.152 |
| 60-69 | 992.9667 | 942.0334 | 1049.509 | 930.5803 | 1065.351 | 920.2192 | 1076.063 | 899.0685 | 1084.685 | 874.0841 | 1088.748 |
| 70-79 | 854.7003 | 813.8763 | 899.4919 | 803.0066 | 915.6193 | 791.7156 | 935.7374 | 776.2358 | 935.7374 | 749.7328 | 937.5481 |
| 80+ | 815.4258 | 755.0467 | 879.5458 | 744.0346 | 895.2449 | 728.3506 | 907.0968 | 713.4334 | 924.0331 | 680.9618 | 934.5198 |
|  |  |  |  |  |  |  |  |  |  |  |  |
| All ages | 222.3856 | 212.2388 | 233.151 | 209.8706 | 236.0078 | 207.2913 | 239.1363 | 203.4364 | 242.2961 | 197.8219 | 243.3567 |

Table 12: Uncertainty analysis around incidence rates in Agincourt sub-district

| DisMod II input and output, database Example | | | | |  |  |  |  |  |  |  |
| --- | --- | --- | --- | --- | --- | --- | --- | --- | --- | --- | --- |
| Uncertainty results, Stroke Incidence (Rates * 100,000), sex: Females | | | | | | |  |  |  |  |  |
| Written 2015/01/25, 01:27:31 PM | | | |  |  |  |  |  |  |  |  |
| Age | Central value (rates * 100000) | Lower (rates * 100000) | Upper (rates * 100000) | Lower (rates * 100000) | Upper (rates * 100000) | Lower (rates * 100000) | Upper (rates * 100000) | Lower (rates * 100000) | Upper (rates * 100000) | Lower (rates * 100000) | Upper (rates * 100000) |
| 0-4 | 0 | 0 | 0 | 0 | 0 | 0 | 0 | 0 | 0 | 0 | 0 |
| 5-14 | 8.334 | 8.1473 | 8.5341 | 8.0858 | 8.5661 | 8.0235 | 8.6547 | 7.9241 | 8.6997 | 7.8535 | 8.7391 |
| 15-29 | 218.1548 | 209.1043 | 227.7929 | 207.3535 | 231.1318 | 204.9564 | 233.5182 | 200.4196 | 237.8484 | 194.553 | 246.5341 |
| 30-44 | 716.5761 | 675.6113 | 762.1474 | 667.8358 | 774.0021 | 661.1696 | 781.5302 | 651.468 | 790.9189 | 630.158 | 794.3594 |
| 45-59 | 565.8346 | 540.713 | 593.5385 | 536.8873 | 601.7795 | 531.1185 | 606.0975 | 522.5315 | 620.5967 | 508.7643 | 628.3351 |
| 60-69 | 916.5916 | 870.3266 | 963.1494 | 860.5355 | 986.6467 | 848.1217 | 1008.164 | 835.4017 | 1023.493 | 814.3992 | 1024.339 |
| 70-79 | 936.0564 | 885.3828 | 1002.942 | 869.6958 | 1011.425 | 852.2927 | 1013.336 | 836.3574 | 1017.084 | 808.0737 | 1020.354 |
| 80+ | 933.6584 | 879.0555 | 996.8913 | 865.8952 | 1001.573 | 849.5166 | 1007.214 | 828.0996 | 1014.235 | 786.2658 | 1018.862 |
|  |  |  |  |  |  |  |  |  |  |  |  |
| All ages | 335.0924 | 318.0726 | 354.0293 | 314.6578 | 359.231 | 310.8394 | 362.7384 | 305.4223 | 368.0569 | 295.9081 | 372.4527 |
